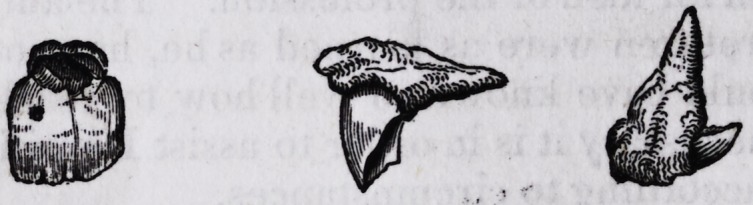# Account of a Remarkable Tooth, with Drawings

**Published:** 1839-06

**Authors:** E. Baker

**Affiliations:** Dentist, New-York.


### ACCOUNT OF A REMARKABLE TOOTH, WITH DRAWINGS,

By E. Baker, Dentist, New-York.

The tooth represented in the figure below, was extracted by me nearly twenty years since, from the mouth of a boy about twelve years of age, in Norfolk, Virginia.

The history of the case was as here represented. The child, when about eight years of age, fell, and this tooth coming in contact with a stone was broken about where the crown of the tooth joins the fang, and in such a manner, that the crown forms a right angle with the fang.

The phenomena attending this case, are, that the broken part of the tooth should be retained in the mouth after the accident, and that a secretion of ossific matter should take place, so as to completely join those two parts together. Having stated the fact, I will not obtrude my opinion concerning the *modus operandi* of nature, in joining those two almost separated parts together, but submit it to more able physiologists.

I may, however, observe, that this case will perhaps entirely settle the conflicting opinions concerning the organization and vitality of the teeth. Dr. Hunter was of opinion, that they were extraneous bodies; that they had no circulation in the bony part, yet possessing a living principle.—Since his time, the opinion has been constantly increasing, that they are not only connected by their organization to the system in general, but have nerves, blood-vessels, and absorbents; and are analagous in this respect, to other bones. That there are in the internal cavity of the teeth, nerves and blood vessels, has not been disputed; but whether these essential components of animal organization, *do* or *do not* enter into the composition of the teeth themselves.

In conclusion: does not the case prove, that the teeth are not only *like* the other bones of the human body, but possess as actively the living and self preservative principle? For it must be recollected, that the healing of the tooth here represented, was accomplished nothwithstanding its exposure to the atmosphere, to the effects of the aliment taken into the mouth and to the danger of disturbing the fractured part by the tongue and other teeth, while in the process of healing.

## Figures and Tables

**Figure f1:**